# A novel *GUCY2D* mutation in a Chinese family with dominant cone dystrophy

**Published:** 2013-05-21

**Authors:** Xin Zhao, YanFan Ren, Xiaohui Zhang, Changxi Chen, Bing Dong, Yang Li

**Affiliations:** Beijing Institute of Ophthalmology, Beijing Tongren Eye Center, Beijing Tongren Hospital, Capital Medical University, Beijing Ophthalmology & Visual Sciences Key Lab. Beijing, China

## Abstract

**Purpose:**

To describe the clinical and genetic findings in a Chinese family with autosomal dominant cone dystrophy (adCOD).

**Methods:**

One family was examined clinically, and genomic DNA was extracted from venous blood of all participants. Genotyping and haplotyping analysis was performed on the known genetic loci for adCOD and autosomal dominant cone-rod dystrophies (adCORD) with a panel of polymorphic markers in this family. All coding exons of the *AIPL1*, *PTTPNM3*, and *GUCY2D* gene were directly sequenced. Allele-specific PCR was used to validate a substitution in all available family members and 100 normal controls. Bioinformatics analysis was done using the Garnier-Osguthorpe-Robson method to predict the effect of the variants detected on the secondary structure of the *GUCY2D* protein.

**Results:**

Clinical examination and pedigree analysis revealed a three-generation family with four members diagnosed with adCOD. Through genotyping, the disease-causing genes were mapped to chromosomes 17p13.1–2 (*AIPL1*, *PITPNM3*, and *GUCY2D* gene). A novel A->G transition at position 2545 (p.T849A) of the cDNA sequence was identified in the *GUCY2D* gene. No mutation was detected in the *AIPL1* and *PITPNM3* genes. This missense mutation co-segregated with the disease phenotype of the family but was not found in the 100 normal controls.

**Conclusions:**

A novel missense mutation of the *GUCY2D* gene was identified in this study. Our results further confirm that the dimerization zone of RetGC-1 is the mutational hot region for COD and CORD.

## Introduction

Inherited cone dystrophies (CODs) and cone-rod dystrophies (CORDs) are a subgroup of inherited retinal degenerative diseases [1]. Characterized by the degeneration of cones with the relative preservation of rod function, CODs cause an early loss of visual acuity and color discrimination in the first decade of life. In contrast, CORDs are characterized by the progressive loss of cone photoreceptor function, followed by the progressive loss of rod photoreceptor function [1]. Both conditions are genetically heterogeneous and can be inherited in autosomal dominant, recessive, or X-linked patterns. To date, 10 genes have been identified as being responsible for adCOD and adCORD, namely, semaphorin 4A (*SEMA4A*) on chromosome 1q22, prominin 1 (*PROM1*) on chromosome 4p15.32, guanylate cyclase activator 1A (*GUCA1A*) and peripherin 2 (*PRPH2*) on chromosome 6p21.1, regulating synaptic membrane exocytosis 1 (*RIMS1*) on chromosome 6p13, guanylate cyclase 2D (*GUCY2D*), arylhydrocarbon receptor interacting protein-like 1 (*AIPL1*), and PITPNM family member 3 (*PITPNM3*) on chromosome 17p13.1–2, unc-119 homolog (*UNC119*) on chromosome 17q11.2, and cone-rod homeobox (*CRX*) on chromosome 19q13.32 (RetNet).

The *GUCY2D* gene, located on chromosome 17p13.1, encodes a 1103 amino acid membrane-bound retinal guanylyl cyclase-1 protein (RetGC-1), which is expressed in both the cone and rod photoreceptors but predominantly in the cone outer segment [2-5]. RetGC-1 is one member of a pair of membrane-bound guanylate cyclases, RetGC-1 and RetGC-2, which synthesize cyclic 3′, 5′-guanosine monophosphate (cGMP) from guanosine triphosphate in mammalian photoreceptor cells. RetGC-1 and its associated activator proteins are responsible for the Ca^2+^-sensitive restoration of cGMP levels after light activation of the phototransduction cascade. RetGC-1 consists of an extracellular or intradiskal domain, a transmembrane segment, a kinase homology domain, a dimerization domain, and a catalytic domain [6]. Heterozygous mutations in the *GUCY2D* gene have been shown to cause adCOD and adCORD [2-4]; however, homozygous or compound heterozygous mutations cause autosomal recessively inherited Leber Congenital Amaurosis (LCA), the most severe form of inherited retinopathy, with total blindness or greatly impaired vision recognized at birth or in early infancy [7,8].

In this study, we investigated a Chinese family with cone dystrophy. After linkage analysis, we mapped the disease-causing gene to regions 17p13.1–17p13.2 where the *GUCY2D, AIPL1*, and *PITPNM3* genes are located and found a novel missense mutation of the *GUCY2D* gene.

## Methods

### Patients and DNA samples collection

This study was performed according to the tenets of the Declaration of Helsinki for research involving human subjects. This study was approved by the Beijing Tongren Hospital Joint Committee on Clinical Investigation. After informed consent was obtained, all participants underwent full ophthalmic examinations, which included bilateral best-corrected visual acuity using E decimal charts, detailed examination of the anterior segment by slit-lamp biomicroscopy, fundus examination with dilated pupils, and a color discrimination test using pseudoisochromatic plates. The proband underwent visual field testing, an electroretinogram, and optical coherence tomography examination.

### Genotyping and haplotyping analysis

Genotyping was performed with 24 microsatellite markers from the autosomes for the known adCOD and adCORD loci in this family (Appendix 1). The fine mapping primer sequences were obtained from the Human Genome Database. Pedigree and haplotype maps were constructed using Cyrillic v. 2.0 software.

### Mutation screening of the *GUCY2D*, *AIPL1*, and *PITPNM3* genes

Mutation screening was performed in the family using direct DNA sequence analysis. All coding regions of the *GUCY2D, AIPL1*, and *PITPNM3* genes were amplified by PCR from the genomic DNA. Primers for all coding exons and exon-intron boundaries of the three genes (18 exons for the *GUCY2D*, 5 exons for the *AIPL1*, and 20 exons for the *PITPNM3*) were designed by the Primer3 program (Appendix 2). For direct sequencing, the PCR products were purified (Shenneng Bocai PCR purification kit; Shenneng, Shanghai, China). The purified PCR products were sequenced using an automatic fluorescence DNA sequencer (ABI, Prism 373A; Perkin Elmer, Foster City, CA) according to the manufacturer’s instructions. All PCR products were sequenced in both forward and reverse directions, and the nucleotide sequences were compared with the published DNA sequences of the *GUCY2D, AIPL1*, and *PITPNM3* genes (GenBank accession number NM_000180, NM_014336, and NM_031220, respectively) using DNAssit version 1.0. For the three genes, cDNA numbering +1 corresponded to A in the ATG translation initiation codon of *GUCY2D*, *AIPL1*, and *PITPNM3*, respectively.

### Allele-specific PCR analysis

To confirm the variation found in the sequencing, allele-specific PCR analysis (AS-PCR) was performed in the available family members and in 100 normal controls [9]. An allele-specific forward primer in Exon 13 of the *GUCY2D* gene was designed: 5′-GGA GCT GGA AAA GCA GAA GG-3′, where C is the mutation-specific nucleotide. The AS-PCR fragment was amplified with the forward allele specific primer and the normal reverse primer of the exon13 of the *GUCY2D* gene.

### Bioinformatics analysis

Garnier-Osguthorpe-Robson software was used to predict the effect of the mutation on the secondary structure of *GUCY2D* (Biotools) [10]. This method infers the secondary structure of a sequence by calculating the probability for each of the four structure classes (helix, sheet, turn, and loop) based on the central residue and its neighbors from the calculated matrices [10]. The PolyPhen2 (Polymorphism Phenotyping 2) program was used to predict the potential functional impact of an amino acid change [11].

## Results

### Clinical findings

**Figure 1:**
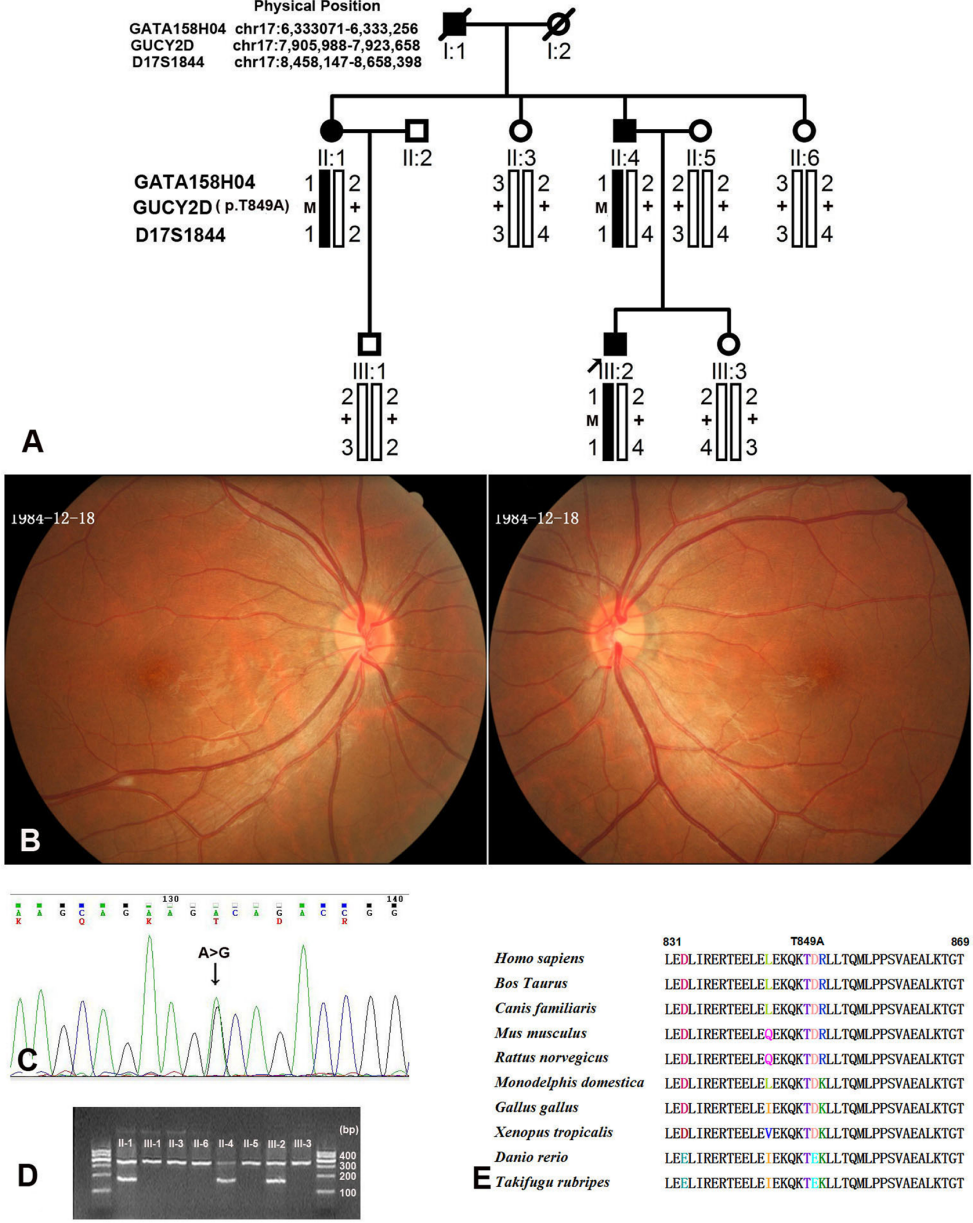
Family structure, proband fundus appearance, DNA sequence chromatograms, and co-segregation analysis of the p.T849A mutation with the disease phenotype in a Chinese family with cone dystrophy. **A**: The pedigree and haplotype analysis of the family with cone dystrophy showed segregation with three microsatellite markers on chromosome 17 listed in rising order from the telomere end. Squares indicate males; circles indicate females; slashed symbols indicate deceased; solid symbols indicate affected; open symbols indicate unaffected; M indicates mutant; and + indicates wild-type. **B**: Fundus appearance of the proband shows the subtle mottling of the RPE in the macula. **C**: Heterozygote sequence (sense strand) shows an A/G transition in codon 849 that changed threonine to alanine. **D**: Allele-specific PCR analysis presents the amplified products of the mutation allele (184 bp) co-segregated with patients in this family. The fragments (325 bp), which are the parts of exon3 of the *MYOC* gene, were used as the internal control in the allele-specific PCR analysis. **E**: The sequence alignment portion of the dimerization domain spanning the p.T849 of the *GUCD2Y* of the human with other species.

**Figure 2:**
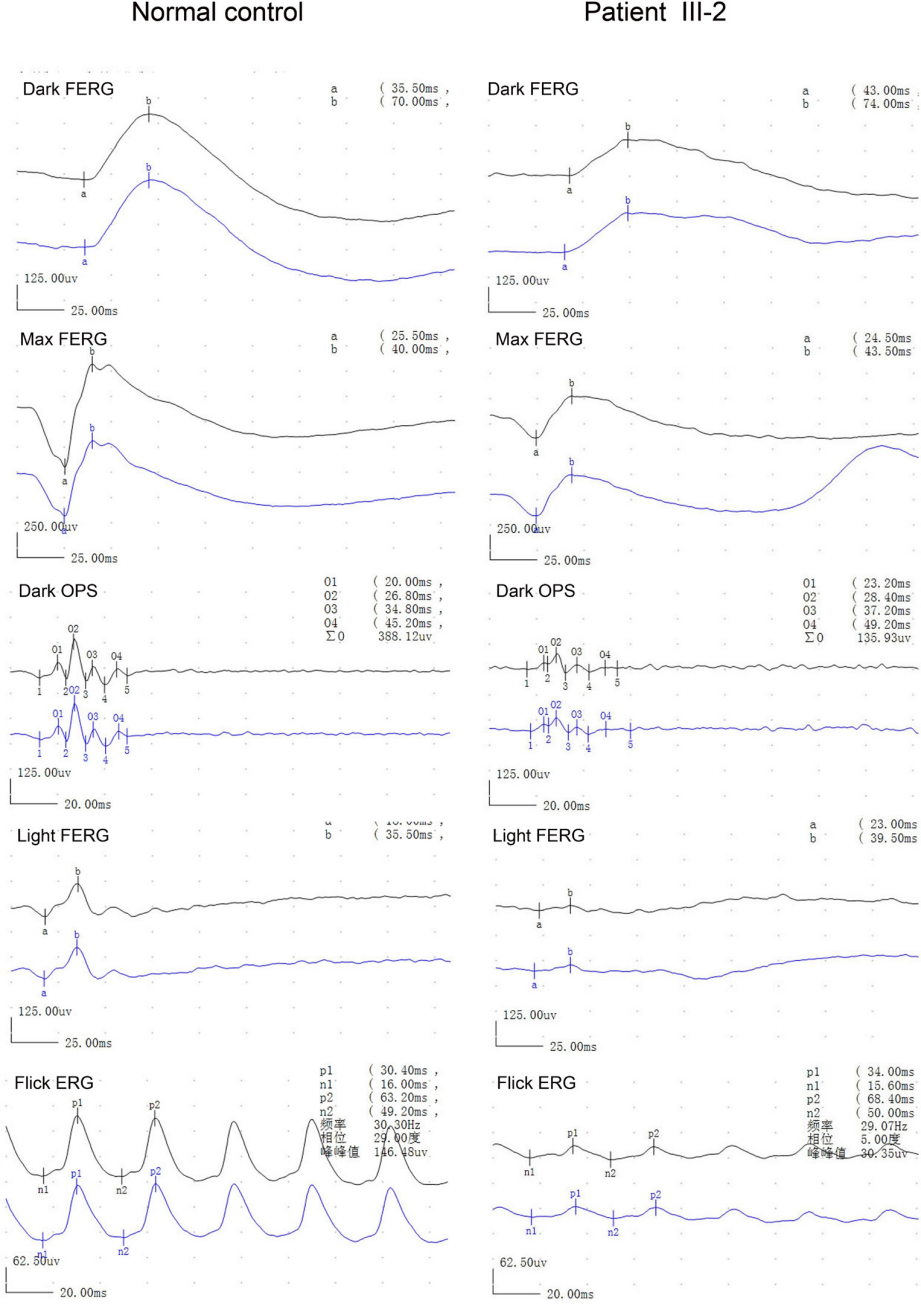
Electroretinography of the proband and a normal control. Electroretinography of the proband shows reduced photopic and 30 Hz responses and normal scotopic responses.

**Figure 3:**
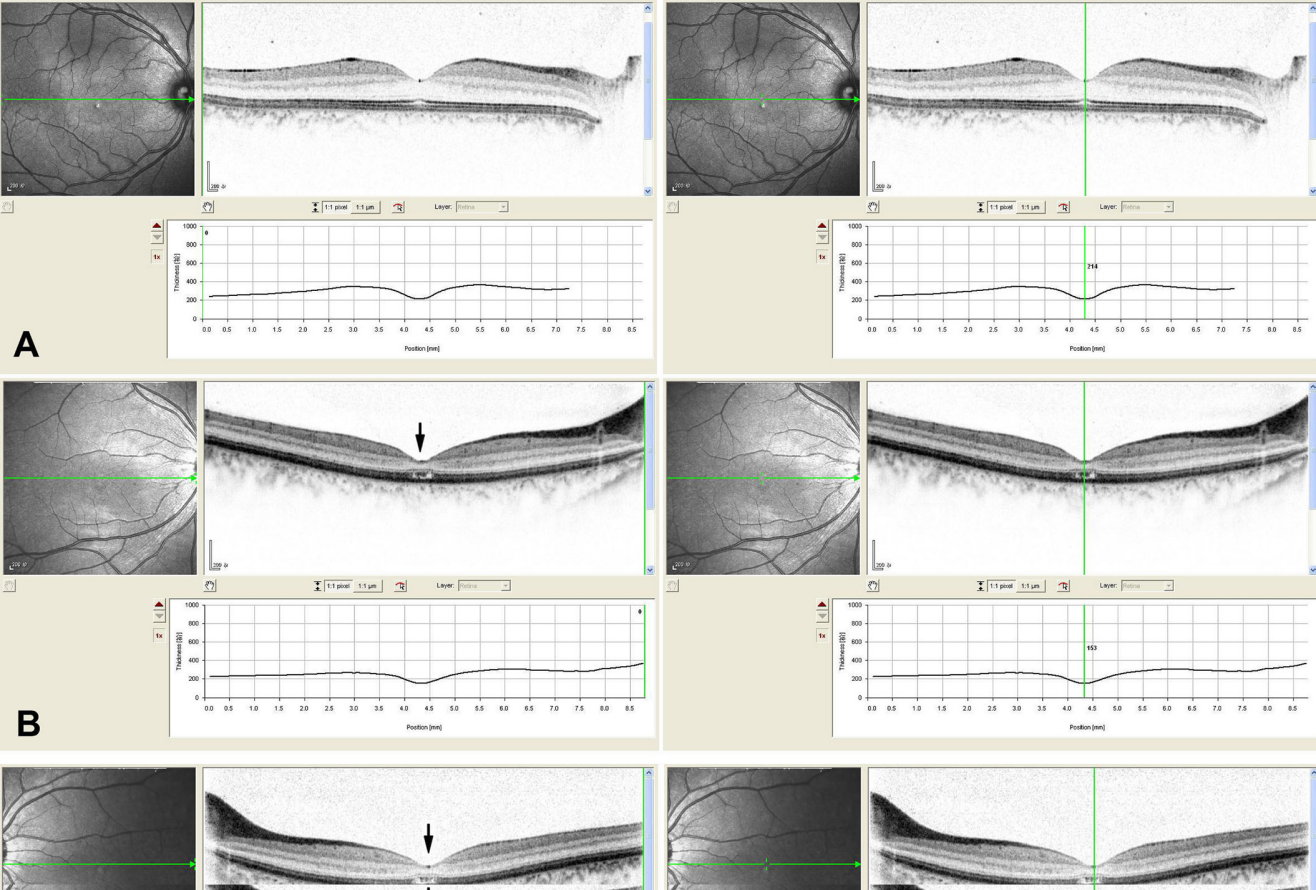
Macular optical coherence tomography images from a visually normal subject and the proband of this family with cone dystrophy. **A**: The macular optical coherence tomography images of the right eye from a normal individual show organization of retinal microstructures with a well defined photoreceptor inner/outer segment layer and normal thickness (214 μm). **B** and **C**: The macular optical coherence tomography images of both eyes from the proband exhibit loss of inner/outer segment layer and thinning of the retina in the macular area (151 μm of the right eye, 153 μm of the left eye).

We identified a three-generation family consisting of four patients diagnosed with cone dystrophy (Figure 1A). All patients had experienced bilateral visual acuity impairment and marked photophobia in their early childhood. No patients, including the two patients aged over 50 years, had peripheral field loss or a nyctalopia complaint. Slit-lamp examination showed the anterior segments were normal with the exception of mild cataracts in both eyes of one patient (II-1). Fundus examinations revealed subtle RPE granular abnormalities in the macular area and normal appearance of the peripheral retina. A pseudoisochromatic plates test showed red-green color weakness. The proband was examined in 2008 at age 24 years and again in 2011 at age 27 years. His best-corrected visual acuity was 0.5 in both eyes, and there was no significant deterioration during the three following years. Fundus examination showed almost normal fundus appearance with the exception of subtle mottling of the RPE in the macular area (Figure 1B). Electroretinographic testing revealed a significant reduction in cone responses and normal rod responses (Figure 2). Optical coherence tomography showed thinning of the retina and loss of the photoreceptor inner segment (IS) and photoreceptor outer segment (OS) in the macular area (Figure 3). The detailed clinical features are summarized in Table 1.

**Table 1:** Clinical features of the patients of this family with adCOD

Patient	Age	Best corrected visual acuity (R/L)	Onset age of photophobia	Night blindess	Refraction (diopters)	Fundus appearance	Color vision	ERG
II-1	56	0.1/0.2	EC	NO	−2.75–1.0X180, −2.25–0.75X180	RPE granular abnormalities at the fovea	red-green defect	N/A
II-4	53	0.1/01	EC	NO	−5.0–1.25X175,-4.5–1.50X180	RPE granular abnormalities at the fovea	red-green defect	N/A
III-6	28	0.5/0.5	EC	NO	−0.5X180,-0.5X175	RPE granular abnormalities at the fovea	red-green defect	reduction in cone responses and normal rod responses

Abbreviations: R, right eye; L, left eye; EC, early childhood, N/A, data not available.

### Genotyping results

This family was genotyped with 24 polymorphic markers around known adCOD and adCORD loci. The mapping results excluded the other known adCOD and adCORD loci with the exception of a locus, 17p13.1–2, where the *GUCY2D*, *AIPL1*, and *PITPNM3* genes are found.

### Mutation analysis

Sequencing of the three genes (*GUCY2D*, *AIPL1*, and *PITPNM3*) revealed one novel heterozygous mutation c.2545 A> G (p.T849A) in the *GUCY2D* gene. Using AS-PCR analysis, this mutation co-segregated with the adCOD phenotype in this family and was not detected in the unaffected members or 100 normal controls (Figure 1C,D).

In addition to the pathogenic mutation detected in the *GUCY2D* gene, six nonpathogenic sequence variants were also identified in this study. Table 2 summarizes these variants based on their nature.

**Table 2:** Presumed nonpathogenic variants found in this study

Gene	Exon	Nucleotide change	Codon	SNP
AIPL1	Exon3	c.276–10 A>C		rs12453262
		c.300A>G	p. L100L	rs8075035
PITPNM3	Exon4	IVS3+56G>T		rs11656015
	Exon6	c.477C>T	p.S159S	rs145362623
GUCY2D	Exon3	c.741C>T	p.H243H	rs3829789
	Exon10	c.2100C>T	p.P700P	rs34598902

### Bioinformatics analysis

Using the Garnier-Osguthorpe-Robson method, the results of the secondary structure prediction suggested that the mutant *GUCY2D* 849A replaced one helix, “H,” with one β sheet, “E,” at position 852. Through PolyPhen-2 program analysis, p.T849A was predicted to be potentially damaging.

## Discussion

In this study, we identified one novel missense mutation, p.T849A, in the *GUCY2D* gene in a small family with adCOD. The mutation co-segregated with the disease phenotype but was absent in the unaffected family members and 100 normal controls.

RetGC-1 is essential for the recovery of the dark state after the excitation process of the photoreceptor cells by light stimulation [6]. To date, more than 120 mutations of the *GUCY2D* gene have been identified as being responsible for retinal degeneration [2-5,7,8,12-23]. Most of them were found in the autosomal recessively inherited LCA [7,8]. The mutations found in COD and CORD were mainly clustered in codon 838 or the two adjacent codons, 837 and 839 [2-5,12-15]. Codon 838 is a mutational hot spot with five disease-causing sequence variations (R → C/G/H/P/S) [2-5,12-15,20]. The most frequent mutations, p.R838C and p.R838H, have been identified in different ethnicities, such as the Caucasian, Spanish, Japanese, and Chinese populations [2-5,12-15,18,22,23]. Unlike the mutations detected in LCA, which are mainly located in the catalytic and kinase-like domains of the RetGC-1 [7,8], most of the mutations identified in COD or CORD are located in the putative dimerization domain, which extends from amino acid 817 to 857 [2-5,12-15,17,18,22,23]. The Thr849 residue located in the dimerization domain is fully conserved in the different species (Figure 1E). The complex missense mutations, p.Q847L and p.K848Q, which were identified in a Japanese family with COD, are just adjacent to the novel mutation p.T849A [17]. Our results further confirm that the dimerization zone of RetGC-1 is the mutational hot region for COD or CORD, and a heterozygous mutation of *GUCY2D* not involving codon 838 can also be linked to COD and CORD.

In the clinical phenotype of the affected members of the family with the mutation p.T849A, the visual acuity of the proband was 0.5 with almost normal fundus. The male subject’s electroretinograms demonstrated reduced cone function and nearly normal rod function. Two elder patients (over 50 years old) had preserved peripheral visual fields and no complaints of night blindness. These findings are similar to the previous descriptions of the phenotypes associated with the mutations p.R838C and p.R838H [2-5]. Since the mutation p.R838C has been identified, the detailed clinical phenotypes with similar or different mutations have been reported by several studies [2-5,14,16,17,21,22]. Usually, patients with the mutations p.R838C and p.R838H have relatively similar clinical features of COD, which include the marked dysfunction of the cones from a young age while rod dysfunction appears later or does not present until a later stage [2-5,15,17,18,22,23].

In conclusion, we identified a novel mutation, p.T849A, in a Chinese family with COD. Our results further suggest that the dimerization zone of RetGC-1 is the mutational hot region for COD or CORD.

## Appendix 1.

Markers used in known autosomal dominant cone dystrophy and autosomal dominant rod dystrophy genotyping. To access the data, click or select the words “Appendix 1.”

## Appendix 2.

Primer information for the *AIPL1*, *PITPNM2*, and *GUCY2D* gene sequence. To access the data, click or select the words “Appendix 2.”
